# Supplementary material for: Investigation of DHA-Induced Regulation of Redox Homeostasis in Retinal Pigment Epithelium Cells through the Combination of Metabolic Imaging and Molecular Biology
Source: Antioxidants (Basel). 2022 May 28;11(6):1072. doi: 10.3390/antiox11061072 (PMC9219962; doi:10.3390/antiox11061072)
Supplement: Supplementary file 1 [file antioxidants-11-01072-s001.zip › antioxidants-1737301-supplementary.pdf]

antioxidants

MDPI

Supplementary Materials

S1. Quantification of Bax and Bcl-2 protein expression

Levels of the pro-apoptotic Bax and anti-apoptotic Bcl-2 protein, respectively, have been evaluated in ARPE-19 cells under different conditions. Results, reported in the graphs in Figure S1, show that in cells treated with high glucose, the treatment with DHA keeps the levels of Bax similar to the untreated control (HG+DHA=0.47±0.01, CTRL=0.52±0.04, p-value = 0.97), and it increased significantly the intracellular levels of Bcl-2 (HG+DHA=2.08±0.12, p-value = 0.004 vs HG) that are instead reduced by the treatment with high glucose concentration (1.34±0.06, p-value = 0.05 vs CTRL=1.70±0.07).

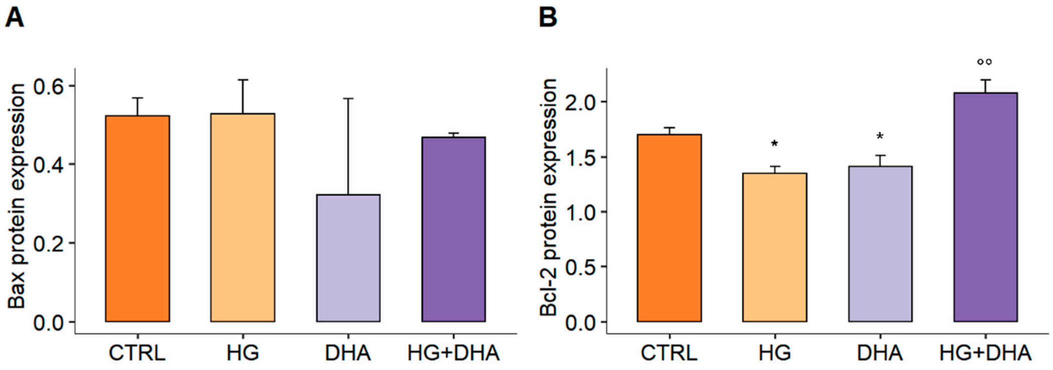

Figure S1. Expression of Bax **(A)** and Bcl-2 **(B)** protein in ARPE–19 cells cultured in physiological condition (5 mM glucose, CTRL, dark orange), with a 50 mM glucose concentration (HG, light orange), supplemented with 60 μM DHA (DHA, light purple), and treated with DHA after HG administration (HG+DHA, dark purple) is reported. Results are presented as mean±sd. Four replicates for each experimental group were considered (* stands for p < 0.05 vs CTRL; °° stands for p-value < 0.01 vs HG alone).

S2. Quantification of Nrf2 protein expression

To evaluate the potential transcriptional activation of Nrf2, we quantified Nrf2 protein expression in whole cells subjected to the different treatments. As evident from the graph reported in Figure S2, a significant increase (p-value = 0.015) was observed in HG cells (+19%) with respect to CTRL. Moreover, the treatment with DHA in high-glucose conditions induced an increase of +11% with respect to untreated cells, revealing the activation of the Nrf2 responsive pathway as a consequence of HG-induced oxidative stress.

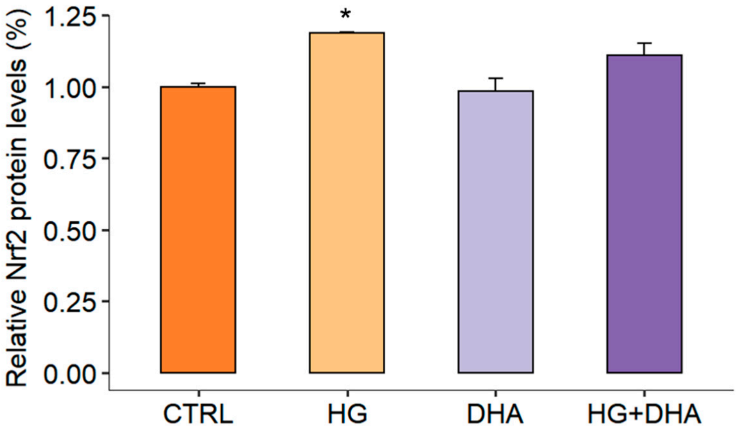

Figure S2. Relative protein levels of Nrf2 in ARPE–19 cells cultured in physiological condition (5 mM glucose, CTRL, dark orange), with a 50 mM glucose concentration (HG, light orange), supplemented with 60 µM DHA (DHA, light purple), and treated with DHA after HG administration (HG+DHA, dark purple) are reported. Results are presented as mean±sd. Four replicates for each experimental group were considered (* stands for $p < 0.05$ vs CTRL).

This measurement provides an indication of Nrf2 translocation from the cytoplasm to nucleus. Indeed, since Nrf2, rather than being ubiquitinated and degraded, is translocated to the nucleus, it is possible to retrieve in an indirect way an increase in the total Nrf2 cellular content. According to recent studies [1], this "whole cell" Nrf2 activation assay, is currently validated when performing experimental treatments that allow comparison against untreated cells, thus avoiding further processing of the cells required to evaluate the contribution from nucleus and cytoplasm separately, as in [2].

References

1. Wasik, U.; Milkiewicz, M.; Kempinska-Podhorodecka, A.; Milkiewicz, P. Protection against Oxidative Stress Mediated by the Nrf2/Keap1 Axis Is Impaired in Primary Biliary Cholangitis. *Sci Rep* **2017**, *7*, 44769, doi:10.1038/srep44769.
2. Clementi, M.E.; Pizzoferrato, M.; Bianchetti, G.; Brancato, A.; Sampaolese, B.; Maulucci, G.; Tringali, G. Cytoprotective Effect of Idebenone through Modulation of the Intrinsic Mitochondrial Pathway of Apoptosis in Human Retinal Pigment Epithelial Cells Exposed to Oxidative Stress Induced by Hydrogen Peroxide. *Biomedicines* **2022**, *10*, 503, doi:10.3390/biomedicines10020503.
